# Influenza (H5N1) Viruses in Poultry, Russian Federation, 2005–2006

**DOI:** 10.3201/eid1304.061266

**Published:** 2007-04

**Authors:** Aleksandr S. Lipatov, Vasily A. Evseenko, Hui-Ling Yen, Anna V. Zaykovskaya, Alexander G. Durimanov, Sergey I. Zolotykh, Sergey V. Netesov, Ilya G. Drozdov, Gennadiy G. Onishchenko, Robert G. Webster, Alexander M. Shestopalov

**Affiliations:** * St Jude Children’s Research Hospital, Memphis, Tennessee, USA; † Federal State Research Institute Research Center for Virology and Biotechnology “Vector,” Koltsovo, Novosibirsk Region, Russian Federation; ‡ Federal Service for Surveillance in Consumer Rights Protection and Human Well-being, Moscow, Russian Federation; 1 These authors contributed equally to this work; 2 Current affiliation: US Department of Agriculture, Athens, Georgia, USA

**Keywords:** Influenza viruses, H5N1, Russia, poultry, pathogenicity, research

## Abstract

We studied 7 influenza (H5N1) viruses isolated from poultry in western Siberia and the European part of the Russian Federation during July 2005–February 2006. Full genome sequences showed high homology to Qinghai-like influenza (H5N1) viruses. Phylogenetic analysis not only showed a close genetic relationship between the H5N1 strains isolated from poultry and wild migratory waterfowls but also suggested genetic reassortment among the analyzed isolates. Analysis of deduced amino acid sequences of the M2 and neuraminidase proteins showed that all isolates are potentially sensitive to currently available antiviral drugs. Pathogenicity testing showed that all studied viruses were highly pathogenic in chickens; for 3 isolates tested in mice and 2 tested in ferrets, pathogenicity was heterogeneous. Pathogenicity in mammalian models was generally correlated with Lys at residue 627 of polymerase basic protein 2.

## Abstract 2

Migrating waterfowl may be the primary source of influenza (H5N1) in western Siberia and the European part of the Russian Federation.

Highly pathogenic avian influenza viruses of the H5N1 subtype are zoonotic agents that present a continuing threat to animal and human health. Before 2003, influenza (H5N1) was endemic in poultry in southern China (*1*,*2*) and occasionally caused severe disease in humans (*2*–*4*). The situation changed in late 2003–2004, when the expanded geographic range of subtype H5N1 resulted in unprecedented epizootics in poultry and new human cases in eastern and southeastern Asia (*5*,*6*). The serious pandemic threat associated with these events intensified the urgency of global pandemic preparedness for influenza (H5N1) (*6*).

In May 2005, an outbreak of influenza (H5N1) in migratory waterfowl was observed at Qinghai Lake in western China (*7*,*8*). Possible spread to Europe by overlapping flyways was a concern (*7*). During 2005–2006, influenza (H5N1) spread throughout Mongolia, Kazakhstan, the Siberian and European part of Russia, Ukraine, countries of the European Union, Africa, and the Middle East (*9*). The first human cases of influenza (H5N1) outside Southeast Asia were reported in 2006 in Azerbaijan, Djibouti, Egypt, Iraq, and Turkey (*9*).

The first influenza (H5N1) epizootics in the Russian Federation occurred at the end of July 2005 in the Novosibirsk region (western Siberia) (*10*–*12*), which borders Kazakhstan and is near Mongolia and northwest China. The outbreaks occurred in backyard poultry flocks and small farms near bodies of water where wild birds presumably stop to feed during seasonal migration. Several studies have reported high sequence homology of all gene segments of influenza (H5N1) isolated in 2005 from wild birds (grebe in Novosibirsk Region and mute swan in Astrakhan Region) and from poultry; these studies also examined the relations of outbreaks in poultry to migrations of wild birds (*12*–*14*). However, evidence that wild migratory birds played a role in the spread of influenza (H5N1) was not conclusive.

After the first outbreaks, influenza (H5N1) spread rapidly westward through Russia; several outbreaks in poultry were reported in western Siberia and south and central European regions of the Russian Federation in late 2005 and early 2006 (Figure 1). At the beginning of March 2006, the influenza (H5N1) epizootics had resulted in the death or slaughter of >1 million poultry in 13 subjects of the Russian Federation. Most of the outbreaks were similar to those first reported in western Siberia (*12*). No human cases of influenza (H5N1) were associated with these outbreaks.

**Figure 1:**
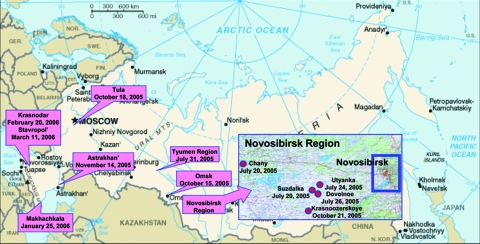
Spread of influenza (H5N1) in the Russian Federation, 2005–2006. Locations and dates of outbreaks of disease in poultry and wild waterfowl (1 outbreak in mute swans, Astrakhan region, Nov 2005) investigated by Federal State Research Institute Research Center for Virology and Biotechnology “Vector.”

Russia lies between eastern Asia and Europe. Surveillance of influenza (H5N1) in poultry and wild waterfowl in these regions could provide unique information about the variety of viruses, their evolution, and possible changes. We characterized 7 influenza (H5N1) viruses isolated from poultry in western Siberia and the European part of the Russian Federation during July 2005–February 2006 (Figure 1, Table 1). Full genome sequences were determined and analyzed, and pathogenicity was determined by inoculation of chickens, mice, and ferrets.

**Table 1:** Influenza (H5N1) viruses, Russian Federation, 2005–2006

Virus	Date of isolation	Type of operation	Specimen used for isolation	IVPI for chickens*
A/chicken/Suzdalka/06/2005	Jul 22, 2005	Backyard flock	Dead chicken (spleen)	3.0
A/goose/Suzdalka/10/2005	Jul 22, 2005	Backyard flock	Dead goose (lungs)	3.0
A/turkey/Suzdalka/12/2005	Jul 22, 2005	Backyard flock	Sick turkey (cloacal swabs)	3.0
A/chicken/Omsk/14/2005	Aug 12, 2005	Backyard flock	Sick chicken (cloacal swabs)	2.6
A/chicken/Tula/4/2005	Oct 5, 2005	Backyard flock	Dead chicken (lungs)	2.8
A/goose/Krasnoozerskoye/627/2005	Oct 17, 2005	Goose farm (~5,000 geese)	Sick goose (cloacal swabs)	3.0
A/chicken/Krasnodar/123/2006	Feb 12, 2006	Chicken farm (~300,000 chickens)	Dead chicken (lungs)	3.0

*IVPI, intravenous pathogenicity index.

## Materials and Methods

### Virus Isolation and Initial Characterization

From July 2005 through March 2006, ≈300 field samples were collected by a research team from the Federal State Research Institute (FSRI) Research Center for Virology and Biotechnology “Vector” (FSRI “Vector”) during 12 outbreaks of influenza (H5N1) in poultry (9 in backyard poultry and 3 at poultry farms) and 1 natural epizootic (mute swans in the Astrakhan Region), ([*14*], Figure 1). Cloacal and tracheal swabs were collected from dead and sick poultry, and internal organs were collected from wild waterfowl found dead near the sites of the outbreaks. Specimen processing and virus isolation were performed at the FSRI “Vector,” a certified Biosafety Level 3 laboratory. Aliquots of field samples (0.1 mL of swab media or of 10% [w/v] organ homogenates) were injected into the allantoic cavity of 10-day-old, specific-pathogen–free embryonated chicken eggs. After incubation at 35ºC for 48 h, the allantoic fluid was harvested, and virus was titrated by hemagglutination test with a 0.5% suspension of chicken red blood cells.

Influenza virus was isolated from 60 samples (20%). The subtype of the hemagglutinin (HA) was determined by hemagglutination inhibition test with 0.5% chicken red blood cells and a panel of antiserum against avian HAs (*15*). The neuraminidase (NA) subtype was determined by NA inhibition assay with a panel of anti-NA serum (*15*). All tested viruses belonged to the H5N1 subtype. Because some samples were duplicated (collected from the same backyard or farm or from birds of the same species with similar disease signs), 36 H5N1 isolates were deposited at the repository of FSRI “Vector.” Because most cases of human H5N1 infection are related to direct contact with infected poultry, we chose 7 poultry isolates for further characterization (Table 1, Figure 1).

Virus-containing allantoic fluid was stored at –80ºC. The infectivity of stock viruses was determined in 10-day-old embryonated chicken eggs by the method of Reed and Muench (*16*) and expressed as the log_10_ 50% egg infective dose (EID_50_)/mL of allantoic fluid.

### Pathogenicity Tests in Chickens

The intravenous virus pathogenicity index (IVPI) of the 7 influenza (H5N1) isolates (Table 1) was determined as described by Capua and Mutinelli (*17*). Infective allantoic fluid was diluted 1:10 in sterile phosphate-buffered saline (PBS), and 0.1 mL was injected intravenously into each of ten 6-week-old, specific-pathogen–free chickens. The chickens were examined for clinical signs of disease once a day for 10 days. Pathogenicity was scored as 0 (no signs of illness), 1 (signs of illness), 2 (signs of severe illness), or 3 (death within 24 h of inoculation). The pathogenicity index was then calculated as the mean score per bird per observation. An index of 3 indicated that all birds died within 24 h; an index of 0 meant that no bird showed signs of illness during the 10-day observation period.

### PCR Amplification and Sequencing

Viral RNA was isolated from virus-containing allantoic fluid with the RNeasy Mini kit (QIAGEN, Valencia, CA, USA) as specified by the manufacturer. Uni12 primer was used for reverse transcription. PCR was performed with a set of primers specific for each gene segment of influenza A virus (*18*). PCR products were purified with the QIAquick PCR purification or QIAquick gel extraction kit (QIAGEN). Sequencing was performed by the Hartwell Center for Bioinformatics and Biotechnology at St Jude Children’s Research Hospital. DNA sequences were completed by using the Lasergene sequence analysis software package (DNAStar, Madison, WI, USA). The nucleotide sequences obtained in this study have been deposited in the GenBank database under accession numbers EF205154–EF205209.

### Phylogenetic Analysis

For phylogenetic analysis, we chose 2 gene segments encoding the main surface antigens (HA, nt 77–1704; NA, nt 21–1349) and 2 conserved genes encoding internal proteins potentially associated with virulence in mammalian species (polymerase basic protein 2 [PB2], nt 58–2304; nonstructural protein [NS], nt 27–855). To identify related reference viruses, we performed nucleotide BLAST analysis of each virus sequence; sequences were uploaded from the Influenza Sequence Database at Los Alamos National Laboratory (www.flu.lanl.gov) (*19*). Sequences were compared by ClustalW alignment algorithm by using BioEdit Sequence Alignment Editor (www.mbio.ncsu.edu/BioEdit/bioedit.html). To estimate phylogenetic relationships, we analyzed nucleotide sequences by the neighbor-joining method with 100 bootstraps by using PHYLIP (the PHYLogeny Inference Package) version 3.65 (http://evolution.gs.washington.edu/phylip.html).

### Pathogenicity Tests in Mice and Ferrets

The 50% mouse lethal dose (MLD_50_), 50% mouse infective dose (MID_50_), and virus titers and organ tropism of 3 influenza (H5N1) isolates were determined for 8-week-old female BALB/c mice. To determine MLD_50_ and MID_50_, we anesthetized groups of 4 mice with diethyl ether (inhalation) and inoculated them intranasally with 50 µL of 10-fold serial dilutions of allantoic fluid in PBS. The mice were observed for death (MLD_50_) for 15 days, or they were killed on day 5 after challenge and tested for pulmonary virus by inoculation of 10-day-old embryonated chicken eggs (MID_50_). MLD_50_ and MID_50_ were calculated by the method of Reed and Muench (*16*). To determine organ tropism, groups of 3 mice were inoculated intranasally with 50 µL PBS containing 10^3^ EID_50_ of virus. In our experience, this viral dose allows the distinction of specific organ tropism among viruses with different pathogenicity patterns in mice. After 5 days, mice were killed and lungs, brain, spleen, liver, and kidneys were collected. Organ homogenates (10% in PBS) were injected into 10-day-old embryonated chicken eggs to detect and titrate virus. Titers were expressed as log_10_ EID_50_/0.1 mg of organ tissue.

The pathogenicity and replication of 2 influenza (H5N1) isolates were characterized in a ferret model. Groups of 3 male 8-month-old outbred ferrets were anesthetized by inhalation of diethyl ether and inoculated intranasally with 10^6^ EID_50_ of virus in 0.5 mL PBS. This inoculation dose is commonly used to characterize the pathogenicity of influenza (H5N1) in this animal model (*20*,*21*). Ferrets were observed for disease signs for 14 days after inoculation; rectal temperature and body weight were measured daily. Nasal washes were collected on days 1–12 as described (*20*,*21*). Virus titers were determined in 10-day-old embryonated chicken eggs and expressed as log_10_ EID_50_/mL of nasal wash fluid.

## Results

### Pathogenicity in Chickens

Groups of 10 chickens were inoculated with the 7 influenza (H5N1) viruses to determine the IVPI index (*17*). Five of the viruses resulted in the deaths of all 10 chickens during the first 24 hours and therefore had an IVPI index of 3 (Table 1). Two isolates, A/chicken/Omsk/14/2005 and A/chicken/Tula/4/2005 viruses, killed all 10 chickens within 48 hours and had IVPI scores of 2.6 and 2.8, respectively (Table 1). All 7 viruses were highly pathogenic (*17*).

### Genetic Characterization

Sequence analysis of PCR products from the 7 isolates demonstrated ≥99% nucleotide identity with the A/bar-headed goose/Qinghai/0510/2005 (H5N1) virus (*7*,*22*) in all gene segments except the NS gene (>98% nucleotide identity). Therefore, all viruses chosen for this study were Qinghai-like influenza (H5N1).

**Figure 2:**
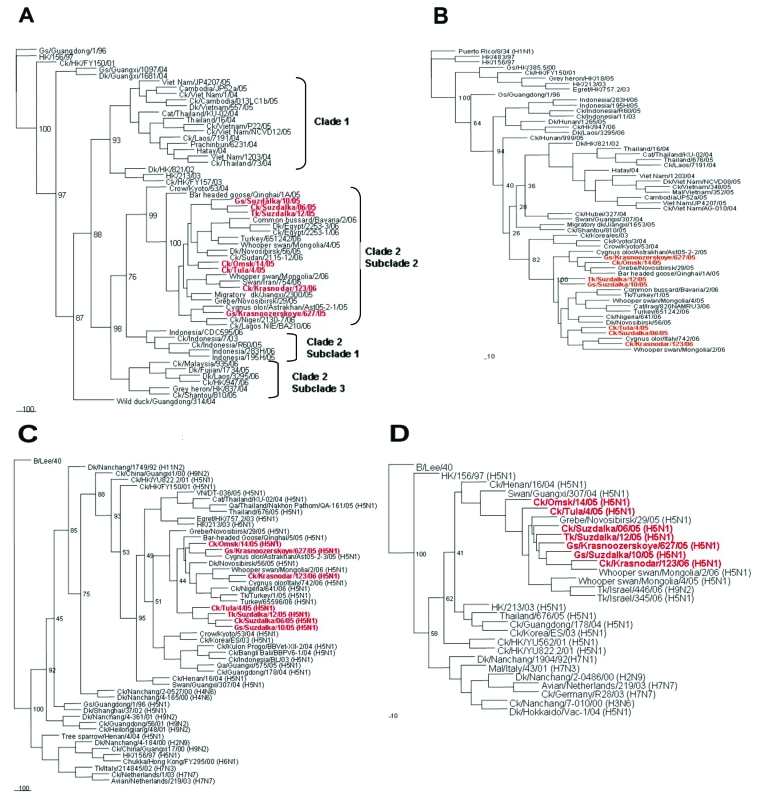
Phylogenetic relationships of the hemagglutinin (HA) (A), neuraminidase (NA) (B), polymerase basic protein 2 (PB2) (C), and nonstructural (NS) (D) genes of the 7 influenza (H5N1) viruses. Nucleotide sequences were analyzed by using the neighbor-joining method with 100 bootstraps. The HA phylogenetic tree was rooted to the HA gene of A/goose/Guangdong/1/96 (H5N1) virus. The NA phylogenetic tree was rooted to the NA gene of Puerto Rico/8/34 (H1N1) virus. The PB2 and NS trees were rooted to the PB2 and NS genes of B/Lee/40 virus.

We performed phylogenetic analysis of the HA, NA, PB2, and NS genes of the 7 influenza (H5N1) isolates with sequences uploaded from the Influenza Sequence Database (*19*) (Figure 2). All studied isolates belonged to subclade 2 of clade 2 of H5 HA (Figure 1A) (*23*). Qinghai-like influenza (H5N1) isolated in Asia, Europe, Africa, and the Middle East are closely related in this HA clade. The goose H5N1 isolate from the October 2005 outbreak in the Novosibirsk Region was phylogenetically closely related to virus isolated from a mute swan (*Cygnus olor*) in November 2005 in the southern European part of Russia (Figure 1) (*14*), and virus isolated from a chicken in February 2006 was phylogenetically closely related to virus isolated in 2006 from a swan in Iran (Table 1, Figure 2A).

The NA, PB2, and NS genes of the isolates (Figure 2B, C, D) were phylogenetically related to those of Qinghai-like viruses. The NA gene of A/chicken/Krasnodar/123/2006 virus was closely related to that of A/Cygnus olor/Italy/742/2006 (Figure 2B); the PB2 genes of A/goose/Krasnoozerskoye/627/2005 and A/chicken/Krasnodar/123/2006 were closely related to those of A/Cygnus olor/Astrakhan/Ast05-2-3/2005 and A/Cygnus olor/Italy/742/2006, respectively (Figure 2C), and the NS gene of A/chicken/Krasnodar/123/2006 was related to that of A/whooper swan/Mongolia/2/2006 virus (Figure 2D). The phylogenetic distribution of the studied isolates differed for each of these genes and for HA. These findings suggested that reassortment events had occurred among the analyzed isolates within the group of Qinghai-like influenza (H5N1) viruses.

The phylogenetic analysis data demonstrated that influenza (H5N1) viruses in poultry and in wild migratory waterfowl are related. These phylogenetic relationships, together with the temporal and geographic correspondence of the poultry outbreaks and the wild waterfowl migratory patterns (*13*,*14*,*24*), support the involvement of wild birds in the perpetuation and spread of Qinghai-like influenza (H5N1). However, until other possible routes of viral dissemination are analyzed and excluded, whether wild migratory birds are the primary source of influenza (H5N1) virus transmission and infection of poultry cannot be conclusively determined.

### Potential Sensitivity to Antiviral Drugs

The H5N1 strains recently isolated in Southeast Asia are resistant to amantadine and rimantadine (*5*,*25*), which target the M2 ion channel protein of influenza A viruses. Influenza (H5N1) viruses resistant to the NA inhibitor oseltamivir have been isolated from oseltamivir-treated patients (*26*,*27*). To determine the potential sensitivity of the studied viruses to these antiviral drugs, we analyzed the amino acid sequences of their M2 and NA proteins.

Amantadine-resistant influenza A variants carry amino acid substitutions at residues 26, 27, 30, 31, or 34 of the M2 protein (*28*,*29*). Our sequence analysis did not show any substitutions at these residues. Therefore, all 7 isolates are potentially sensitive to this class of antiviral drugs.

Amino acid residues 119, 274, 292, and 294 of the NA protein (numbered according to NA of the N2 subtype) are crucial for sensitivity to NA inhibitors (*30*); the substitutions H_274_→Y and N_294_→S were reported to confer resistance to oseltamivir in clinical influenza (H5N1) isolates (*26*,*27*). No amino acid substitutions were observed at the conserved residues in the NA protein of the studied viruses, which suggests that they are sensitive to NA inhibitors.

### Molecular Correlates of Pathogenicity in Mammals

The receptor specificity of the HA protein could be crucial for efficient replication and spread of a pandemic strain (*31*). In the HA molecules of all 7 viruses, amino acid residues relevant to receptor binding retained the 2,3-NeuAcGal linkages predicted to confer affinity for avian cell surface receptors (*5*,*32*). A multibasic cleavage site in the H5N1 HA is essential for lethal infection in a mouse model (*33*). We found all 7 isolates contained the multibasic amino acid motif PQGERRRKKR/GL (characteristic of Qinghai-like viruses) at their HA cleavage sites.

Residues in the viral polymerase complex (PB1, PB2, and polymerase acidic protein [PA]) may be associated with the adaptation and virulence of avian viruses in mammals (*33*–*36*). Sequence analysis of these proteins revealed Lys_627_ in the PB2 of 3 studied isolates: A/chicken/Omsk/14/2005, A/goose/Krasnoozerskoye/627/2005, and A/chicken/Krasnodar/123/2006. In mice, influenza (H5N1) viruses with Lys_627_ are highly virulent and replicate systemically (*33*). Other residues associated with adaptation and virulence, i.e., residues 701 of PB2 (*34*,*35*), 13 of PB1 (*35*), and 615 of PA (*35*), were those typical of avian viruses with low virulence in mammals.

Analysis of NS1, which may also contribute to the virulence of influenza (H5N1), showed a deletion of 5 amino acids that is similar to that found in genotype-Z influenza (H5N1) viruses and that may contribute to increased expression of tumor necrosis factor-α and interferon-γ–inducible protein 10 (IP-10) in primary human macrophages (*2*). No viruses contained Glu_92_ in the NS1, which is associated with the high virulence of H5N1 subtype in 1997 (*37*,*38*), and all contained the “avian-like” PDZ-domain ligand ESEV (*39*).

### Pathogenicity and Replication in Mice and Ferrets

The pathogenicity and organ tropism of the 3 influenza (H5N1) isolates A/turkey/Suzdalka/12/2005, A/goose/Krasnoozerskoye/627/2005, and A/chicken/Krasnodar/123/2006 were characterized in a mouse model (Table 2). Of these viruses (isolated in 2005), 2 had a substitution at residue 627 in PB2 that is associated with pathogenicity in mice (*33*); we chose A/chicken/Krasnodar/123/2006 because it was the only virus isolated in 2006. Isolate A/goose/Krasnoozerskoye/627/2005 was highly pathogenic and replicated systemically in mice. A/chicken/Krasnodar/123/2006 virus had MID_50_ and MLD_50_ values similar to those of A/goose/Krasnoozerskoye/627/2005 virus but was recovered only from mouse lungs, where it replicated to lower titers (Table 2). Virus A/turkey/Suzdalka/12/2005 replicated efficiently in the brains and lungs of mice, although the MID_50_ and MLD_50_ values of this virus indicated low pathogenicity. In general, these data agreed with the results of sequence analysis: the PB2 proteins of both highly pathogenic viruses contained Lys_627_, which confers high virulence in mice (*33*). However, which molecular determinants restricted the replication of A/chicken/Krasnodar/123/2006 virus to the lungs remains to be determined.

**Table 2:** Pathogenicity and replication of influenza (H5N1) viruses in mice*

Virus	Log_10_ EID_50_/mL	MID_50_^†^	MLD_50_^†^	Titers in mouse organs^‡^				
				Lungs	Spleen	Brain	Liver	Kidney
A/goose/Krasnoozerskoye/627/2005	9.2	10^2.2^	10^2.3^	6.1±0.3	1.6±0.5	5.2±0.2	1.6±0.3	2.6±0.2
A/turkey/Suzdalka/12/2005	9.3	10^5.3^	10^6.3^	4.1±0.6	<1	2.3±0.5	<1	<1
A/chicken/Krasnodar/123/2006	8.4	10^2.1^	10^2.3^	4.7±0.3	<1	<1	<1	<1

*EID_50_, 50% egg infectious dose; MID_50_, 50% mouse infectious dose; MLD_50_, 50% mouse lethal dose. †The 50% mouse infective dose (MID_50_) and mouse lethal dose (MLD_50_) are expressed as number of EID_50_. Values are the means of 2 titration experiments. ^‡^Mean ± SD from 3 mice, expressed as log_10_ EID_50_/0.1 mg of organ tissue.

**Figure 3:**
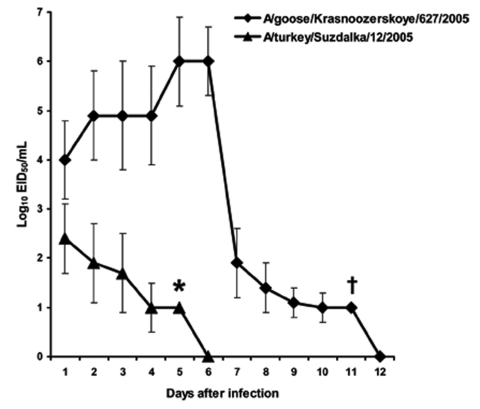
Replication of influenza (H5N1) viruses in ferret upper respiratory tract. Male 8-month-old outbred ferrets were inoculated intranasally with 10^6^ 50% egg infectious dose (EID_50_) of virus in 0.5 mL phosphate-buffered saline. Virus titers are the mean ± SD from 3 ferrets, expressed as log_10_ EID_50_/mL of nasal wash fluid. *Virus was detected in 2 ferrets. †Virus was detected in 1 ferret.

Because the 2 isolates from 2005 (A/turkey/Suzdalka/12/2005 and A/goose/Krasnoozerskoye/627/2005) showed distinct pathogenicity in mice, they were further characterized in the ferret model. The results of pathogenicity studies in ferrets were consistent with those in mice. A/goose/Krasnoozerskoye/627/2005 caused severe disease accompanied by respiratory and neurologic signs previously described in ferrets inoculated with influenza (H5N1) (*20*,*21*). All inoculated ferrets had fever on days 1–7 after inoculation and had substantial weight loss (as much as 23%, data not shown). This virus replicated at high titers in the upper respiratory tract and was recovered from nasal washes until day 11 after inoculation (Figure 3). A/turkey/Suzdalka/12/2005 virus demonstrated low pathogenicity in ferrets. Temperature elevation was observed on days 1 and 2 after inoculation, but no other disease signs or substantial weight loss were noted (data not shown). The virus replicated at low titers in the upper respiratory tract, reached peak titers on day 1 after inoculation, and was cleared by day 6 (Figure 3). The molecular differences between the A/goose/Krasnoozerskoye/627/2005 and A/turkey/Suzdalka/12/2005 viruses are shown in Table 3. The 2 viruses differed most in their polymerase proteins. On the basis of available data about the molecular determinants of pathogenicity of influenza (H5N1) in ferrets (*20*,*21*,*36*), we propose that some of these residues underlie the observed differences in pathogenicity.

**Table 3:** Molecular differences between influenza (H5N1) isolates with high and low pathogenicity in mammalian models

Protein*	Position	Virus	
		A/turkey/Suzdalka/12/2005	A/goose/Krasnoozerskoye/627/2005
PB2	80	I	T
	473	L	M
	483	M	V
	627	E	K
	666	T	I
PB1	654	S	G
	655	M	I
	744	L	M
PA	377	S	N
	604	R	K
	693	A	V
HA	2	K	E
	8	L	F
	170	N	D
	289	N	S
NA	60	V	L
	266	G	S
NP	389	K	R

*PB, polymerase basic protein; PA, polymerase acidic protein; HA, hemagglutinin; NA, neuraminidase; NP, nucleoprotein.

## Discussion

Our findings demonstrate that the influenza (H5N1) viruses isolated from poultry in Russia are Qinghai-like influenza (H5N1) viruses (*22*) and are phylogenetically related to viruses isolated from wild migratory waterfowl (Figure 2). Phylogenetic analysis of these poultry isolates supports the possibility that genetic reassortment had occurred among the Qinghai-like viruses. Kilpatrick and coauthors, in a recent study of the global spread of influenza (H5N1), proposed that influenza (H5N1) viruses were likely introduced into Russia from China by migrating birds and that wild migrating birds play a role in spreading influenza (H5N1) into Europe (*40*). Collectively, our genetic findings, the rapid dissemination of viruses over great distances (Figure 1), and the apparent correspondence between migratory patterns and the sites and timing of poultry outbreaks (*24*) indicate a correlation but do not prove conclusively that wild migrating birds are the primary source of influenza (H5N1) infection of poultry in Russia. Analysis of a greater number of viruses isolated from poultry and wild birds, epidemiologic studies in affected areas, and characterization of other possible human-related modes of virus dissemination and transmission (i.e., trade of poultry or poultry products, spread via rail and motor vehicle routes) might provide confirmatory data.

The studied viruses were highly pathogenic in chickens, but their pathogenicity was heterogeneous in mouse and ferret animal models. The pattern of pathogenicity we observed was generally correlated with known molecular determinants of influenza (H5N1) pathogenicity in mammals.

Influenza (H5N1) outbreaks in poultry in the Novosibirsk Region have caused the deaths of 5,031 birds and the slaughter of 93,620 (a 19% loss) (*12*). In the Russian Federation as a whole, >1 million birds were lost during influenza (H5N1) epizootics from July 2005 through March 2006. Several control measures have been undertaken to prevent the spread of influenza (H5N1) in poultry and potential transmission to humans (*12*). The first is slaughter and disposal of sick poultry and other birds in close contact with them. The second is quarantine of villages and poultry farms where influenza (H5N1) infection is confirmed or suspected. These measures include restriction of the movement of any poultry or poultry products and disinfection of all affected facilities and of any vehicles entering and exiting the area. The third is sanitary and veterinary measures at poultry farms and in backyard flocks in the affected regions to prevent contact of poultry with wild birds and the potential spread of virus by vehicles. The regional spread of influenza (H5N1) and outbreaks at the main poultry production facilities have been halted. No human cases have been reported during or since the 2005–2006 epizootics; therefore, these measures appear to have been effective. February 2006 saw the start of vaccination of poultry at farms and in backyard flocks in the affected areas with inactivated whole-virus influenza (H5N1) vaccines. At present, the effectiveness of the vaccination campaign cannot be assessed.
